# Dual Targeting of EGFR and MTOR Pathways Inhibits Glioblastoma Growth by Modulating the Tumor Microenvironment

**DOI:** 10.3390/cells12040547

**Published:** 2023-02-08

**Authors:** Maxim Sidorov, Pratiksha Dighe, Rinette W. L. Woo, Aida Rodriguez-Brotons, Michelle Chen, Ryan J. Ice, Edith Vaquero, Damon Jian, Pierre-Yves Desprez, Mehdi Nosrati, Leah Galvez, Lewis Leng, Lawrence Dickinson, Mohammed Kashani-Sabet, Sean David McAllister, Liliana Soroceanu

**Affiliations:** 1 California Pacific Medical Center Research Institute, 475 Brannan St, Suite 130, San Francisco, CA 94107, USA; 2 Pacific Brain and Spine Medical Group, Eden Medical Center-Sutter Research, 20103 Lake Chabot Rd, Castro Valley, CA 94546, USA

**Keywords:** glioblastoma, erlotinib, MLN0128, tumor-associated macrophages, periostin

## Abstract

Glioblastoma’s (GBM) aggressive growth is driven by redundant activation of a myriad of signaling pathways and genomic alterations in tyrosine kinase receptors, such as epidermal growth factor receptor (*EGFR*), which is altered in over 50% of cases. Single agents targeting EGFR have not proven effective against GBM. In this study, we aimed to identify an effective anti-tumor regimen using pharmacogenomic testing of patient-derived GBM samples, in culture and in vivo. High-throughput pharmacological screens of ten EGFR-driven GBM samples identified the combination of erlotinib (EGFRi) and MLN0128 (a mammalian target of rapamycin inhibitor, or MTORi) as the most effective at inhibiting tumor cell viability. The anti-tumor activity of erlonitib+MLN0128 was synergistic and produced inhibition of the p-EGFR, mitogen-activated protein kinase (MAPK), and Phosphoinositide 3-kinase (PI3K) pathways in culture. Using an orthotopic murine model of GBM, we show that erlotinib+MLN0128 inhibited tumor growth in vivo and significantly prolonged the survival of tumor-bearing mice. Expression profiling of tumor tissues from treated mice revealed a unique gene signature induced by erlotinib+MLN0128, consisting of downregulation of immunosuppressive chemokines in the tumor microenvironment, including C-C motif chemokine ligand 2 (CCL2) and periostin. Lower periostin levels resulted in the inhibition of Iba1+ (tumor-promoting) macrophage infiltration of GBM xenografts. Taken together, our results demonstrate that pharmacological co-targeting of EGFR and MTOR using clinically available drugs represents an effective treatment paradigm for EGFR-driven GBMs, acting both by inhibiting tumor cell growth and modulating the immune tumor microenvironment.

## 1. Introduction

Glioblastoma (GBM) represents the most common and deadly type of primary brain tumor in adults. In spite of advances in surgery, radiation, and chemotherapy, GBMs remain very difficult to treat, with an average five-year survival post-diagnosis at 12% [1,2]. Over 80% of GBMs are the *IDH1*-wild type and are driven by alterations in receptor tyrosine kinases (RTK), as well as the MAPK/RAS and PI3K/AKT/MTOR pathways [3,4,5]. Approximately 50% of GBMs are characterized by activating mutations, amplification, and overexpression of *EGFR*, which subsequently activate downstream signaling pathways, including the PI3K/AKT/MTOR pathway [6,7]. Given its critical role in driving disease progression in GBM, EGFR became an attractive therapeutic target. However, therapeutic resistance due to target loss (EGFR protein) or activation of compensatory mechanisms (e.g., c-MET, IGF1R) has been widely documented [8]. Although targeting activating mutations in EGFR has positively impacted the outcome of patients with colorectal or lung cancer [9,10], the use of such inhibitors as single agents has not shown clinical benefits for GBM [11]. An evaluation of the efficacy of erlotinib (used at 150 mg/d) in relapsed GBM patients identified an overall response rate of 6.9% [12]. A subsequent single-agent erlotinib clinical trial for relapsed GBM patients with confirmed *EGFR* alterations (including EGFRvIII) resulted in a median survival of 7 months which was considered safe, but not effective [13]. A later phase II trial combining erlotinib, radiation, temozolomide, and bevacizumab in previously untreated GBM patients found the median overall survival was at 19 months compared to 18 months for historical controls treated at the same institution [14]. Most recently, erlotinib was included as one of the treatment arms in a genomics-informed feasibility study for recurrent or progressive GBM, where efficacy was not assessed [15]. Taken together, this clinical evidence suggests that subsets of GBM patients may benefit from erlotinib-based treatment regimens, however, better combinatorial strategies are needed. Targeting of the MTOR pathway with single agents has not demonstrated efficacy against GBM, to date [16]. Dual TORC kinase inhibitors were designed to overcome some of the limitations of the first-generation PI3K-AKT-MTOR inhibitors (e.g., Rapamycin). Among these, MLN0128 was shown to sensitize pediatric gliomas to radiation therapy in preclinical models [17]. Next, MLN0128 was used in a clinical trial in combination with Bevacizumab in the setting of recurrent GBM, where minimal anti-tumor activity was noted [18]. A dose escalation trial aimed at finding the recommended phase 2 dose for MLN0128, when combined with bevacizumab in GBM patients, reported stable disease in six out of 16 patients, which was a modest therapeutic effect [19]. As in the case of erlotinib, these clinical trials suggest that a genomics-driven selection of eligible patients and additional combinatorial strategies may be required for MLN0128 to be effective in GBM. Intra-tumor molecular heterogeneity [20] in conjunction with an immunosuppressive tumor microenvironment (TME) are among the factors contributing to therapeutic resistance and disease recurrence in GBM [21]. Therefore, an effective anti-GBM therapy would have to target both tumor intrinsic and TME-dependent tumor-promoting pathways.

In the current study, we set out to identify and test individualized treatment regimens for EGFR-driven GBM, using patient-derived cells (PDC) and patient-derived xenografts (PDX), guided by the samples’ genomic profiles and focusing on small molecule-based therapeutics. We tested a number of small molecule inhibitors with evidence of blood–brain barrier penetrance using high throughput drug screening (HTDS), as previously described by our group [22]. The best-performing drug combination was then subjected to in vivo validation studies using an orthotopic murine model of GBM. The anti-tumor efficacy of the “winning” combination of EGFR (erlotinib) and MTOR (MLN0128) inhibitors was further mechanistically investigated in culture and in vivo.

## 2. Methods

Ethics statement: All patients included in the study were enrolled and consented to the study using an IRB-approved non-treatment protocol (Sutter IRB# 25.125-2). All animal studies were pursued in accordance with a CPMC-approved IACUC protocol (protocol # 18.08.03).

### 2.1. Generation of GBM PDXs and the U937 Cell Line

Patient-derived tumor cells (PDCs): upon receiving human GBM tissue from the operating room, samples were mechanically disrupted and cultured as previously described by our group [23,24,25]. Patient-derived xenograft (PDX) and PDX cultures (PDXC) generation and in vivo passaging were performed as previously described [25]. All PDX and PDC lines used in this study have been authenticated as human and unique, using the ATCC STR profiling service. The U937 line was purchased from ATCC (Catalog # CRL-1593.2) and grown in RPMI media plus 10% FBS as recommended by the manufacturer.

### 2.2. High-Throughput Drug Screening

#### 2.2.1. Set-Up for Pharmacological Screens

All PDXCs or PDCs were plated in 384-well low attachment round-bottom microplates (Corning, Tewksbury, MA, USA) within 3–7 days after processing the original tumor material or thawing frozen cells, and they were then allowed to acclimate for 3 days. The low attachment round-bottom microplates, media conditions, and acclimation period allowed for the formation of tumorspheres before the addition of drugs. GBM PDXCs were screened in an HTDS format against a 6-point concentration-response curve of drugs chosen primarily based on the following criteria: (1) The drug is FDA-approved or in clinical trials; (2) The drug is available for research purposes; (3) Pharmacokinetic (PK) data in humans are available. The highest concentration was set equal to the maximum plasma concentration (C_max_) reported in published clinical trials.

#### 2.2.2. Quantification of Drug Response

Quantification of drug response was performed as previously described by our group [22]. The drug-response data were fitted using area under the pharmacological curve (AUC) and with the program Prism (GraphPad, San Diego, CA, USA) using the formula ∆X*(Y1 + Y2)/2. Past studies and a recent large-scale screening effort demonstrate that AUC is more effective than either calculating the potency and efficacy of a drug across multiple cancer cell lines, since AUC takes into account both drug potency and efficacy in a single value. For ease of evaluation, we used a standard min-max normalization equation to create a scoring scale similar to a typical grading scale. We transformed the AUC value to equal a value between 0–100: score = 100 × (1 − (AUC − AUC_min_/AUC_max_ − AUC_min_), where 0 is no effect and 100 represents the killing of all the cells. Drugs that stimulate cell growth produce a score < 0. As part of our drug scoring platform, all the analyses are automated using specialized in-house VBA (visual basic for application) programmed Excel spreadsheets and Prism. To test for synergism, the combination index (CI) was calculated where CI < 1, =1, and >1 indicate synergism, additive effect, and antagonism, respectively [26]. The fraction affected represents the percentage of cells killed (e.g., 0.2 = 20%) by each of the drug combinations evaluated.

### 2.3. Phospho-Kinase Array

GBM PDXCs plated in 60 mm well plates at a density of 2.5 × 10^5^ cells per well were treated with a vehicle, 20 μM erlotinib, 1 μM MLN0128, or the combination of the two drugs for 24 h. Two hundred µg of total protein from each treatment group was added to the array membranes and the proteome profiler assay was performed according to the manufacturer’s instructions (#ARY003C; R&D Systems). The relative amounts of proteins were quantified using densitometry and the software program ImageJ (NIH).

### 2.4. Western Blot

PDX GBM cells plated in 60 mm well plates at a density of 2.5 × 10^5^ cells per well were treated with erlotinib (20 μM), MLN0128 (1 μM), or a combination of erlotinib and MLN0128 for 24 h. For the control group, cells were treated with DMSO. Cells were then harvested, washed with ice-cold PBS, and lysed in RIPA buffer (#89900; Thermo Fisher Scientific, Waltham, MA, USA) containing protease (#87786; Thermo Fisher Scientific) and phosphatase inhibitor cocktails (#78420; Thermo Fisher Scientific). The cell extracts were fractionated by SDS-PAGE using pre-cast 4–20% gels (#4561094DC; Bio-Rad, Hercules, CA, USA) under reducing conditions and transferred to a polyvinylidene difluoride membrane using a transfer apparatus according to the manufacturer’s protocols (Bio-Rad). Membranes were blocked with 5% non-fat dry milk or BSA in TBST (20 mmol/L Tris pH 7.5, 150 mmol/L NaCl, 0.1% Tween 20) for 1 h at room temperature and then incubated with primary antibodies (See Table 1 below) at 4 °C overnight. Membranes were washed with TBST and incubated with horseradish peroxidase-conjugated anti-mouse (#7076S; Cell Signaling, Danvers, MA, USA) or anti-rabbit (#7074S; Cell Signaling) secondary antibodies for 1 h at room temperature. Blots were washed with TBST and developed with the Clarity Western ECL system (#1705060; Bio-Rad) or SuperSignal West Femto (#34094, Thermo Fisher Scientific) according to the manufacturer’s protocols.

### 2.5. Apoptosis Assays

Caspase 3/7 assays were performed by using the Muse Oxidative Stress Kit, Muse Cell Cycle Kit, Muse Annexin V Apoptosis Kit, and Muse Caspase 3/7 Kit, respectively (EMD Millipore, Hayward, CA, USA) following the manufacturer’s instructions and as described previously [27,28]. GBM192 and GBM218 tumorspheres were treated with erlotinib (20 μm), MLN0128 (1 μm), or the combination, and apoptosis was measured 48 h after the drug treatment. The Muse^®^ Caspase-3/7 Kit allows for the rapid and quantitative measurements of apoptotic status based on Caspase-3.7 activation and cellular plasma membrane permeabilization and cell death. The Muse^®^ Caspase-3/7 Kit simultaneously determines the count and percentage of cells in various stages of apoptosis based on the activity of executioner caspases namely, Caspase-3/7 activity in combination with a dead cell dye.

### 2.6. In Vivo GBM Model and Treatment Study

Three hundred thousand GBM192-derived cells were intracranially implanted in nude mice, as previously described by our group [23]. Twenty-one days following tumor implantation, mice were randomized into 4 treatment groups. Vehicle (8 mice, DMSO), erlotinib (10 mice, 20 mg/kg, QD, 5 days/week), MLN0128 (10 mice, 1 mg/kg; QD; 5 days/week), and the combination of the two drugs (14 mice, same doses as above) were administered i.p. daily for 5 days/week. Mice were monitored closely, in accordance with CPMC IACUC-approved protocols, checking for any signs of neurological decline throughout the experiment, and were euthanized based on the IACUC-approved oncological studies guidelines (>20% body weight loss, hunch posture, poor overall body condition).

### 2.7. RNA Analysis Using Microarray

RNA extraction: Mouse brains were harvested and snap-frozen for downstream analysis. Selected tissues were partially thawed and mashed to create tissue slurries that were then processed using the Qiagen RNA extraction kit.

Data availability: The microarray data associated with this study are freely available on the NCBI site, under accession number GSE178147. Data were analyzed by PhalanxBio as follows: GPR files were loaded into RosettaResolver^®^ System (RosettaBiosoftware, Seattle, WA, USA) to process data analysis. Rosetta profile error model calculation: the error due to random factors and systematic biases is estimated by an error model. Differentially expressed gene lists: standard selection criteria to identify differentially expressed genes are as follows: (1) log2 |Fold change| ≥ 1 and *p* < 0.05. (2) log2 ratios = “NA” and the differences of intensity between the two samples ≥1000.

Clustering analysis: Gene clustering by average linkage algorithm was performed on selected differentially expressed gene lists after data transformation and mean centering. Pathway and GO analysis: After the microarray data were analyzed using the Rosetta software a processed intensity value table was then further manipulated to generate the heatmap. Briefly, the fluorescence intensity data were sorted based on max–min within each gene. The differences were then sorted in descending order and the top genes were selected with a pre-determined numerical cutoff for the number of top probes to be used for heatmap generation. Additional pathway enrichment analyses were performed using the open-source software g-Profiler (https://bio.tools/gprofile, accessed on 6 April 2022).

### 2.8. Taqman Analysis

The same RNA samples as those used for microarray profiling were subjected to Taqman validation of gene expression. A total of 1 μg of eluted total RNA was loaded into each cDNA conversion. The reactions were set up using the parameters stated in the Biorad iScript Advanced Kit manual. Each RT-PCR reaction contained cDNA derived from approximately 20 ng of total RNA from each sample. Analysis was carried out using the ddCt method with error propagation. RNA extracted was reverse transcribed to single-stranded cDNA using the iScript cDNA synthesis kit (# 170-8891; Bio-Rad). Real-time PCR TaqMan gene expression assays (Thermo Fisher Scientific) were used to analyze mRNA expression levels in a ViiA^TM^ real-time PCR system. All reactions were performed in a 96-well plate, in triplicate, with the amplification reaction containing 5.5 μL of TaqMan Fast Advanced Master Mix (#4444557; Thermo Fisher Scientific), 0.5 μL of Taqman primer/probe, 1.8 μL of cDNA template, and 3.5 μL of nuclease-free water. The thermal profile consisted of 50 °C for 2 min, followed by 95 °C for 10 min, and 40 cycles of 95 °C for 15 s and 60 °C for 1 min. Fluorescence was measured once per cycle at the end of the 60 °C segment. The Taqman probes used were RAB14 (Hs00249440_m1), CCL2 (Hs00234140_m1), CXCR4 (Hs00976734_m1), POSTN (Hs01566750_m1), EPHB2 (Hs00362096_m1), and EGFR (Hs01076078_m1). CT (threshold cycle) values of the genes of interest were averaged and calculated relative to the CT values of RAB14 using the 2^−ΔΔCT^ method.

### 2.9. Immunohistochemistry

Immunohistochemistry (IHC) was performed on paraffin-embedded brain tissues collected from treated mice. Hematoxylin and eosin staining was performed to confirm the presence of tumor cells in the brain tissue sections. For IHC, the sections were treated with citrate buffer, pH 6.0 (#C9999; Sigma-Aldrich, St. Louis, MO, USA) for 11 min at 110 °C in a steamer for heat-induced antigen retrieval. 3,3-Diaminobenzidine (DAB) and IHC staining of these sections was then performed according to the manufacturer’s instructions for a series of antibodies shown below, using the super sensitive poly-HRP IHC detection system (QD400-60KE, BioGenex, Fremont, CA, USA). Negative control staining was performed by omitting the addition of primary antibodies. The IHC-stained slides were mounted using the Vectashield HardSet anti-fade mounting medium. The primary antibodies used were Iba1 (Abcam, Fremont, CA, USA- ab178846; 1:200), POSTN (Abcam, ab14041, 1:100), EGFR (Abcam, ab231; 1:150), and Ki-67 (Invitrogen, Waltham, MA, USA MA5-14520; 1:100). Slides were scanned using the Mirax-Midi (from Zeiss, Dublin, CA, USA) whole slide high-resolution scanning system.

### 2.10. ELISA for Human Periostin and CCL2

PDXC from patient-derived GBM192 and GBM218 were plated in 60 mm well plates at a density of 2 × 10^5^ cells per well and were treated with a vehicle, 20 µM erlotinib, 1 µM MLN0128, or a combination of erlotinib and MLN0128 for a total of 48 h. The supernatant from each treatment group was assayed for levels of human CCL2 as per the manufacturer’s instructions using the Human CCL2/MCP-1 Quantikine ELISA Kit (#DCP00; R&D Systems). The relative amounts of proteins were calculated by determining the optical density of each sample using a microplate reader set to 450 nm.

### 2.11. Transwell Migration Assays

U937 cells were plated in a 12-well plate at a density of 5 × 10^5^ cells per well in RPMI 1640 medium with 10% FBS for 24 h before priming. These were primed with 100 nM PMA (Sigma, St Louis, MO, USA; P8139) for 48 h to become monocyte-derived macrophages. Transwell assays assessing cell migration potential were performed on 24-well plates with 8.0 μm pore size PET membrane inserts (Corning BioCoat control insert) according to the manufacturer’s instructions. Briefly, 5 × 10^5^ primed U937 macrophages suspended in 100 μL of serum-free media were seeded and cultured in the upper chamber. Conditioned medium from GBM192 and GBM218 treated with erlotinib (20 μM), MLN0128 (1 μM), or the combination of the two drugs, was added to the lower receiver chamber. The cells were allowed to migrate for 20 h, at the end of which they were fixed with 4% formaldehyde for 2 min, permeabilized with methanol for 20 min, and stained with 0.1% crystal violet for 10 min. After wiping off the upper layer of non-migrated cells with a cotton swab, cells were counted by light microscopy.

## 3. Results

### 3.1. HTDS Identify the Combination of Erlotinib and MLN0128 as Most Effective and Synergistically Inhibiting the Growth of EGFR-Driven GBMs

**Figure 1:**
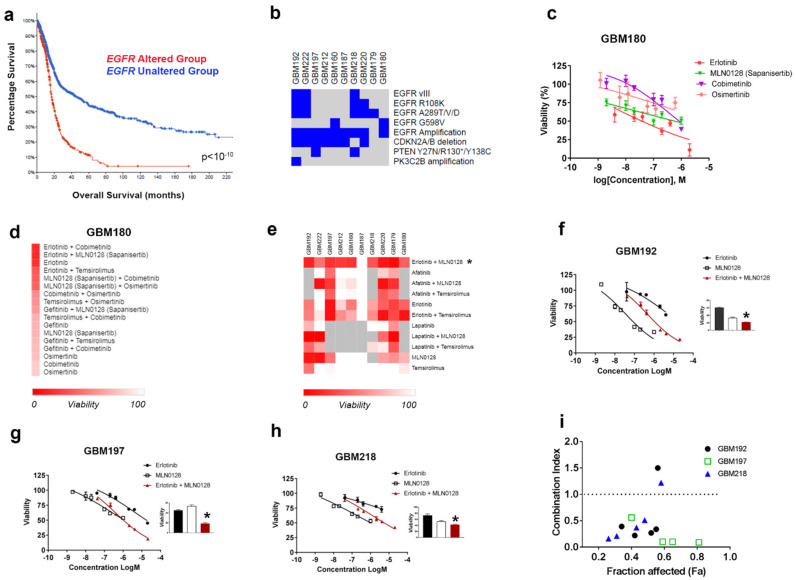
Response of EGFR-driven GBMs to combinatorial targeted therapy. (**a**) Kaplan–Meier survival curves in GBM patients with (red line) or without (blue) *EGFR* alterations (TCGA database). (**b**) Summary of genomic alteration in the subset of GBM samples used for functional studies in this manuscript; blue squares indicate the presence of the alterations listed on the right side of the diagram. (**c**) Full-dose response in GBM 180 treated with 4 targeted therapies, as indicated. (**d**) Heatmap representing the ranking of anti-tumor efficacy for all drugs and combinations used against GBM180. (**e**) Heatmap representing response to drug combinations across the ten GBM samples listed on top of the heatmap (red denotes maximum response; grey not tested). (**f**–**h**) Complete dose-response curves, representing cell viability changes in the presence of erlotinib, MLN0128, and the combination of the two drugs in GBM192 (**f**), GBM197 (**g**), and GBM218 (**h**). Inset graphs show anti-tumor efficacy at 10% Cmax. * *p* < 0.05 two-way ANOVA. (**i**) Combination index values across 6 concentrations for erlotinib+MLN0128 in 3 distinct samples (GBM192, GBM197, GBM218) are shown. The fraction affected represents the percentage of cells killed by each of the drug combinations evaluated. Calculations were performed using Compusyn software. All values below 1 represent synergistic interactions between the drugs.

Through a translational oncology research program at our institution, we screened over 40 GBM PDC, or PDXC (PDX-derived cells) samples using single drugs targeting core signaling pathways in GBM. We found that no single agent is effective at inhibiting tumor cell growth under serum-free (neurosphere) conditions (unpublished observation). We therefore focused instead on identifying drug combinations to target GBM growth. We selected a subset of PDC and PDXCs derived from tumors driven by genomic alterations of *EGFR*-which are reported in over 50% of GBM samples [29]. Table S1 shows the clinical and molecular annotations for the primary-derived GBM samples used in the current study. The rationale for choosing this cohort of GBMs is twofold: first, the poor survival of patients with EGFR alterations as shown in Figure 1a (median survival is 14.3 months vs. 15.1 months for GBM patients with EGFR alterations and EGFR wild type, respectively). Secondly, single-agent EGFR inhibitors have not proved effective in the clinic, as detailed in our introduction and [30]. The main genomic alterations in the 10 GBM samples used in this study are shown in the diagram presented in Figure 1b. We used HTDS to test the effects of EGFR, PI3K-MTOR, and MEK inhibitors on cell viability. As an example, the GBM180-derived culture was subjected to HTDS using a number of inhibitors (Table S2). GBM180 cells (500 cells/well) were plated in a 384-well plate and allowed to form tumorspheres. Seventy-two hours after initial plating, a number of drugs and combinations were dispensed using an acoustic liquid Echo handler as previously published by our group [22]. Cell viability was read 72 h later using Cell Titer Glo. Each drug was tested using six doses. A strategy of our HTDS assays was to set the highest concentration used in the assay to the C_max_ reported for each of the drugs in clinical trials. Evaluating drug exposures in HTDS that are relevant to those observed in clinical practice can improve translation to the clinic. Furthermore, the use of tumorspheres more closely reflects the three-dimensional tumor environment. Full concentration-response curves were run for each drug alone, and the drugs were then combined at their C_max_ and 10% C_max_. Including two concentrations for drug combination analysis controls against false positives and allows further ranking of drug effects to favor drug combinations that produce the greatest effects at the lowest concentration (10% C_max_). Figure 1c shows GBM180 cell viability in response to four individual drugs: erlotinib, MLN0128, cobimetinib, and osimertinib (full-dose responses). Figure 1d shows a heatmap corresponding to the ranking of individual drugs and combinations thereof based on their anti-tumor efficacy (score) when used at 10% Cmax. The ranking scores used to generate the heatmap in Figure 1d are shown in Table S3.

Next, we used the same strategy as described above for GBM180 to perform HTDS (using C_max_ and 10% C_max_ for each of the drugs) across all 10 EGFR-driven GBM samples included in Table S1 (and also shown in Figure 1b). The ranking of treatments identified the combination of erlotinib and MLN0128 as the top-performing combination across all samples (Figure 1e). MLN0128 is also known as *Sapanisertib*, and is currently in clinical trials for lung cancer (NCT044250545). Ranking scores for the drugs and combinations shown in Figure 1e are found in Table S4.

We next investigated whether the combination of erlotinib and MLN0128 may be synergistic in inhibiting GBM cell viability over multiple concentration ratios when combining both drugs. We subjected three EGFR-driven GBM PDX-C lines (i.e., GBM192, GBM197, and GBM218) to 72 h viability assays in 96 well plates using full-dose response for the single drugs and the combination of the two (erlotinib+MLN0128). The complete dose-response curves for GBM192, GBM197, and GBM218 treated with erlotinib and MLN0128 are shown in Figure 1f–h.

Drug efficacy for single drugs and drug combination is ranked by the ability to inhibit cell proliferation and by combination index (CI) values, where a CI value of <1, 1, and >1 indicates synergism, additivity, and antagonism, respectively [31].

The synergy between erlotinib and MLN0128 was documented in all three GBM samples tested in this manner, as shown in Figure 1i, where the CI is below 1 for most drug concentrations used.

### 3.2. Erlotinib and MLN0128 Impact EGFR-Driven GBM Cell Survival by Inhibiting the Tumor-Promoting Pathways p-EGFR, MAPK, and PI3K/AKT/MTOR and Inducing Apoptosis

**Figure 2:**
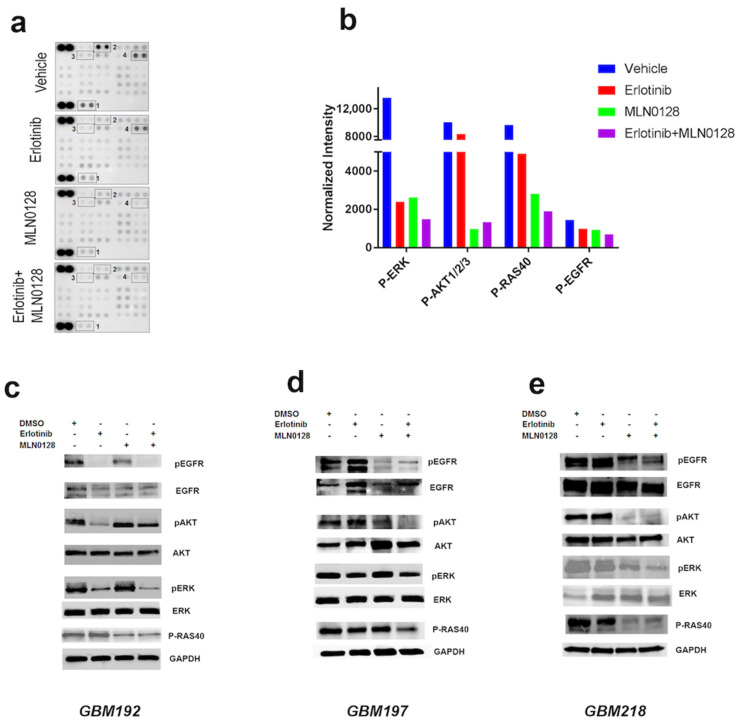
Erlotinib and MLN0128 treatment synergistically impacts growth-promoting pathways in EGFR-driven GBMs. (**a**) Antibody array with 45 cellular phosphor-kinases was used to detect pathways impacted by erlotinib and MLN0128. GBM192 cells were treated with erlotinib (20 μM), MLN0128 (1 μM), or the combination of the two drugs for 24 h. The arrays were scanned and quantified, using the control (darkest spots) for normalization. *1*-p-ERK; *2*-p-AKT, *3*-p-Ras40, *4*-p-EGFR. (**b**) Quantification of the levels of phosphokinases under each treatment condition. Units represent normalized densitometry values. (**c**–**e**) Western blot analyses of the same phosphoproteins were performed in PDXCs from GBM192, GBM197, and GBM218 treated with vehicle (DMSO), erlotinib, MLN0128, or the combination of the two drugs and probed with the indicated antibodies. GAPDH was used to control for equal protein loading.

To investigate the mechanisms underlying the synergistic anti-tumor activity of erlotinib and MLN0128, we interrogated 42 cellular phosphokinases using a phosphor-array from R&D, as previously described by our group [32]. Cell lysates from GBM192 cells treated with the vehicle, erlotinib (20 μM), MLN0128 (1 μM), or the combination of the two for 24 h were hybridized overnight with the phosphor-antibody array. Following signal detection, normalization, and quantification of phosphorylation levels, we detected the downregulation of a number of growth-promoting phosphokinases under drug treatment compared to the control. Included among these were phosphor-ERK, phosphor-AKT, p-RAS40, and phosphor-EGFR (Figure 2a). The quantification of phosphoprotein levels is shown in Figure 2b. In agreement with the pharmacology data, we show that the combination of erlotinib and MLN0128 inhibited p-ERK, p-RAS40, and p-EGFR levels more effectively than either drug alone; phosphor-AKT was equally downregulated by MLN0128 and the erlotinib+MLN0128 combination (Figure 2b). We next set out to validate the array results and interrogate the same signaling pathways in the same three samples where pharmacological synergy was demonstrated for erlotinib and MLN0128. PDXC from GBM192, GBM197, and GBM218 were subjected to Western blot analyses. Results are presented in Figure 2c–e. The combination of erlotinib and MLN0128 more robustly inhibited p-EGFR, p-ERK, and p-RAS40 than either drug alone in GBM192 in concordance with the phosphor-array data (Figure 2c). In GBM192, the lowest levels of p-AKT were detected in the erlotinib treatment alone, which may be in part due to the antibody used which measures specifically Ser473 phosphorylation levels (Figure 2c) compared to cumulative p-AKT levels shown in the phosphor-array (Figure 2b). Of note, while erlotinib was not as effective at inhibiting p-EGFR, p-ERK, and p-AKT in the other two GBM PDXs tested, the combination of erlotinib and MLN0128 profoundly inhibited p-EGFR, p-AKT, p-ERK, and p-RAS40 levels in both GBM197 (Figure 2d) and GBM218 (Figure 2e). Western blot quantification corresponding to Figure 2c–e is shown in Figure S1.

We next interrogated one recurrent GBM sample, GBM212, which is recurrent for GBM197 (same patient). Data for GBM212 is shown in Figure S2. Our results demonstrate that the combination of erlotinib and MLN0128 was more effective at inhibiting tumor cell viability than either drug alone, albeit requiring higher doses than in the case of the matched primary GBM197 (Figure S2a and Table S4). Western blot analyses of GBM212 show that while p-EGFR and p-AKT levels were inhibited, p-ERK was upregulated by the dual therapy (Figure S2b,c). These results are in agreement with the notion that recurrent GBMs become more treatment-resistant to targeted agents, regardless of the frontline therapy administered for the primary disease [33].

To further characterize the anti-tumor activity of erlotinib and MLN0128, we performed apoptosis Caspase 3/7 assays measuring both early and late apoptosis in GBM192 and GBM218 PDXCs (Figure S3a,b). Compared to the vehicle treatment, erlotinib alone induced 12% and 19% apoptosis while MLN0128 induced 14% and 23% apoptosis in GBM192 and GBM 218 respectively (Figure S3). As shown in Figure S3b, the combination of erlotinib and MLN0128 was more effective, inducing 20% and 30% apoptosis in GBM192 and GBM218 respectively. In all cases, these percentages refer to the total apoptosis levels (early + late).

### 3.3. The Combination of Erlotinib and MLN0128 Prolongs the Survival of GBM-Bearing Mice

Next, we wanted to validate the anti-tumor efficacy of erlotinib and MLN018 in vivo, using an orthotopic mouse model of glioma (GBM192 intracranially implanted in nude mice, as detailed in the methods section). In vivo validation of the HTDS findings is critical both for demonstrating the robustness of our drug testing platform, as well as for understanding additional anti-tumor mechanisms which can only be interrogated using an in vivo system.

**Figure 3:**
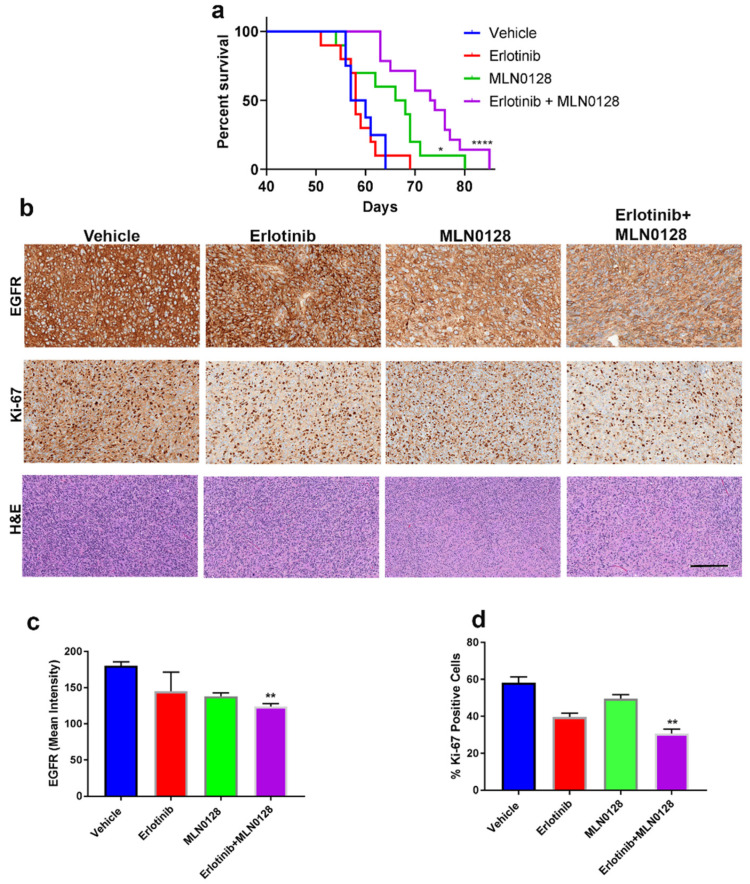
The combination of erlotinib and MLN0128 significantly inhibits GBM growth in vivo, in an orthotopic murine model of glioma. (**a**) Kaplan–Meier survival analysis of mice bearing intracranial GBM192 derived gliomas and treated with either drug alone or the combination of erlotinib and MLN0128. The treatment schedule is detailed in the methods section. * *p* = 0.02; **** *p* < 0.0001 (log-rank Mantel–Cox test). (**b**) Representative photomicrographs of immunohistochemical detection of EGFR (upper panels) and Ki-67 (middle panels) in GBM192 PDX samples from mice treated as indicated. Lower panels represent H&E staining for the same groups. Bar = 150 μm. (**c**) Quantification of EGFR intensity levels in the four different groups. Four low-magnification fields/sections were counted. We used three sections/mouse and three mice/group. (**d**) Ki-67 quantification was performed as described for EGFR, counting positive nuclei and calculating % positive from total nuclei. ** One-way ANOVA *p* < 0.05 (combination vs. vehicle).

Figure 3a shows the Kaplan–Meier survival analysis for all four treatment groups. Vehicle-treated mice had a median survival of 54 days and erlotinib treatment alone did not significantly prolong survival in glioma-bearing mice. MLN0128 treatment alone improved survival (median survival = 65) compared to the vehicle (*p* = 0.001 log-rank test). Importantly, the most profound survival improvement was accomplished by the combination of erlotinib and MLN0128, with a median survival = 74 days (*p* < 0.0001 log-rank test compared to the vehicle). The combination was also significantly better compared to either drug alone (Figure 3a).

Figure 3b shows the immunohistochemical (IHC) evaluation of GBM tumors treated in vivo. This analysis was performed using sequentially cut tissue sections (5 μm apart) and the shown antibodies. EGFR levels are known to be regulated in a compensatory fashion by inhibition of its receptor tyrosine kinase targeted by erlotinib [34] which is consistent with the staining pattern shown in Figure 3b upper panels. IHC analysis of tumor proliferation index as measured by Ki-67 is shown in Figure 3b-middle panels. H&E staining is shown in Figure 3b lower panel.

Quantification of the EGFR IHC staining (Figure 3c) demonstrates that prolonged erlotinib treatment did not induce a significant change in EGFR levels, while chronic treatment with either an MTOR inhibitor (MLN0128) or the combination of MLN0128 with erlotinib significantly inhibited EGFR expression levels for the duration of the study. Quantification of Ki67 levels demonstrates that tumor cell proliferation in vivo was significantly reduced by all drug regimens, albeit most effectively by the dual therapy, erlotinib+MLN0128 (Figure 3d).

### 3.4. Transcriptomic Analyses of GBM Treated In Vivo Reveal Modulation of the Tumor Immune Microenvironment by Erlotinib and MLN0128

We next interrogated the molecular basis of the synergistic tumor growth inhibition exhibited by the combination of erlotinib and MLN018 in vivo. To that end, we used RNA microarray profiling of tumor samples from treated mice. RNA samples were processed at Phalanx bio according to their published protocols https://www.phalanxbiotech.com/, accessed 20 May 2018. Additional details on the microarray hybridization protocol and data analysis are included in the methods section and accompanying the GSE178147 data packet freely available at NCBI-GEO.

**Figure 4:**
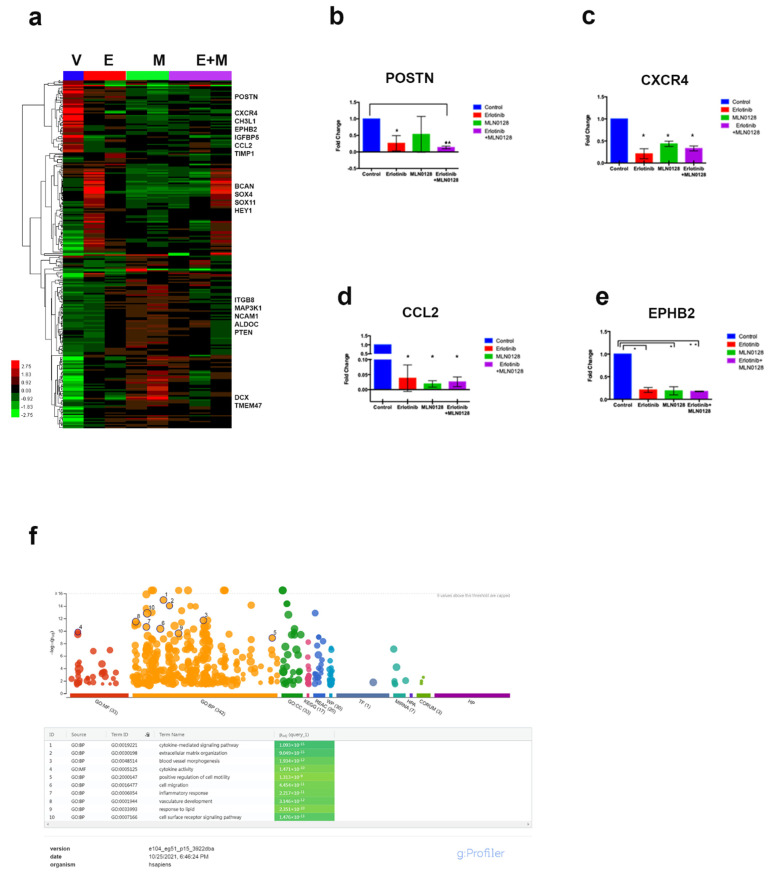
Transcriptomic profiling of GBM192 PDX treated with erlotinib and MLN0128. (**a**) Heatmap showing differentially expressed genes in the vehicle (V), erlotinib (E), MLN018 (M), and combination (E+M) treated tumors. A subset of 200 up- and down-regulated genes (>1.5 fold change; adjusted *p* < 0.05) are shown. On the right side of the heatmap, representative gene names are indicated for each cluster. (**b**–**e**) Taqman validation of microarray data; fold change in expression levels for four genes (POSTN, CXCR4, CCL2, and EphB2) in each treatment group are shown. Statistical analysis was performed using *t*-test. (**b**) * *p* < 0.05; ** *p* < 0.0001, compared to vehicle. (**c**,**d**) * *p* < 0.02 for all treatment arms compared to vehicle. (**e**) * *p* < 0.02; ** *p* < 0.0001 compared to vehicle. (**f**) GO pathways were significantly impacted by the combination of erlotinib+MLN0128 treatment in vivo. The diagram was obtained using the open-source g-Profiler software.

After subtracting the expression of mouse transcripts, differential human gene expression (fold change > 1.5, *p* < 0.05) is displayed in the heatmap shown in Figure 4a. Table S5 shows a list of all differentially regulated genes. The heatmap shows distinct clusters of genes modulated by the targeted therapy. The first cluster includes chemokines and chemokine receptors with documented pro-tumorigenic, immunosuppressive, and pro-inflammatory effects in GBM, such as periostin (POSTN), CXCR4, CH3L1, CCL2, and EphB2 [35]. The dual therapy of erlotinib+MLN0128 was most effective in inhibiting the expression of the above-mentioned genes. Taqman validation for a subset of these genes is shown in Figure 4b–e. The second cluster shown on the heatmap includes genes upregulated by the dual therapy and by erlotinib alone (in one tumor sample), compared to the vehicle. These genes, such as BCAN, SOX11, and HEY1, are associated with the “proneural” GBM molecular subclass and their expression is correlated with a less aggressive phenotype [36]. The third cluster shown on the right side of the heatmap includes another set of downregulated genes, which are involved in tumor metabolism and survival (ALDOC, PTEN) and tumor cell adhesion or motility (ITGB8, NCAM1). Inputting the list of significantly differentially expressed genes between the combo therapy group and the vehicle in the open-source g-Profiler program (https://bio.tools/gprofiler, accessed 6 April 2022) we obtained a list of enriched gene ontology (GO) pathways, the top ten of which (ranked by statistical significance) are shown in Figure 4f. Among the significantly impacted pathways, cytokine-mediated signaling, inflammatory response, extracellular matrix organization, and cell migration are noted. Taken together, these analyses suggest that gene expression changes induced by erlotinib+MLN0128 in vivo, are concordant with improved survival in this treatment group.

Interestingly, among the cytokines shown in the first cluster, POSTN and CCL2 were most robustly downregulated in GBM samples treated with the combination of erlotinib and MLN0128. We subsequently interrogated the functional implication of this inhibitory effect, as detailed below.

### 3.5. Combinatorial Therapy Using Erlotinib and MLN0128 Downregulates POSTN and CCL2 Levels and Inhibits the Iba1-Positive Macrophages in the GBM TME

POSTN is a potent chemoattractant for Iba1-positive “M2” phenotype macrophages (or microglia) in the GBM microenvironment, where they exhibit pro-tumorigenic activity [37,38]. CCL2 is also involved in driving a pro-tumorigenic, inflammatory phenotype in GBM, especially in cases where EGFR and EGFRvIII cross-activation is present [39]. Interestingly, GBM192 shows alterations in wild-type EGFR as well as expression of EGFRvIII (Table S1). We next focused on better understanding how pharmacological inhibition of these two cytokines (POSTN and CCL2) modulate the GBM microenvironment in vivo and in culture.

**Figure 5:**
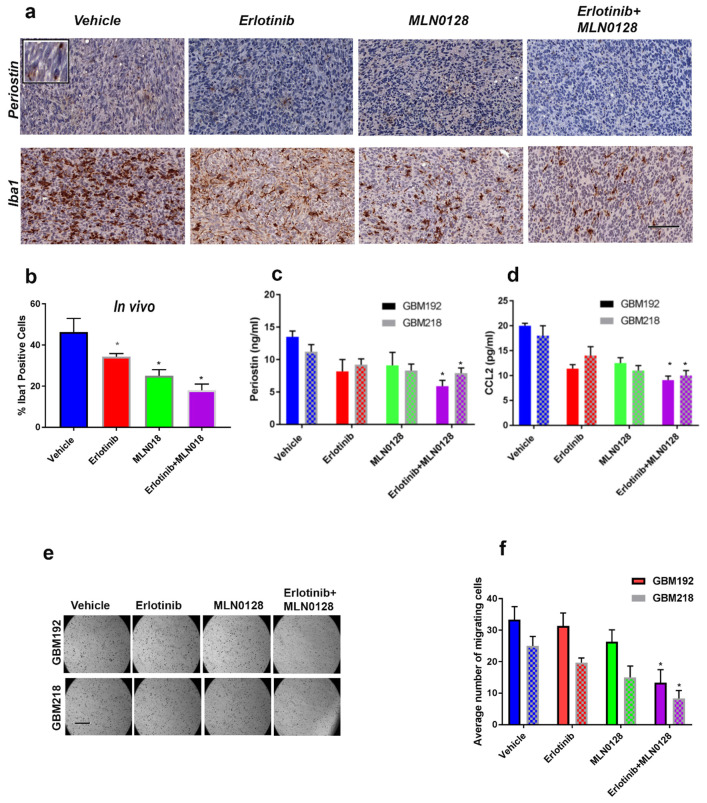
Combinatorial therapy using erlotinib and MLN0128 downregulates POSTN and CCL2 levels and inhibits the Iba1-positive macrophages in the GBM TME. (**a**) POSTN (upper panels) and Iba1 (lower panels) immunohistochemical staining of GBM PDX samples from mice treated as indicated. Representative photomicrographs are shown. Bar = 150 μm. (**b**) Quantification of Iba1 positive cells in GBM192 treated xenografts was performed by counting 6 low-magnification fields in 2 sections/mouse; 3 mice/treatment group. * *p* < 0.05 for each treatment compared to vehicle (*t*-test). (**c**) ELISA detection of POSTN in GBM192 (solid bars) and GBM218 (patterned bars) cultured over 48 h in the absence of growth factors and treated with inhibitors erlotinib 20 μM and MLN0128 1 μM. * *p* < 0.05 comparing dual therapy to vehicle. The experiment was repeated three times and data from a representative experiment are shown. (**d**) ELISA measurements for CCL2 in supernatant from GBM192 (solid bars) and GBM218 (patterned bars) cultured over 48 h in the absence of growth factors and treated with inhibitors as shown. * *p* < 0.05 dual therapy compared to vehicle. The experiment was repeated three times and results from a representative experiment are shown. (**e**) Supernatants from GBM192 (upper panels) and GBM218 (lower panels) treated as explained for c–d were used to induce chemotaxis of U937 cells in a transwell migration assay. Representative photomicrographs are shown. Bar = 250 μm. (**f**) Quantification of migrating cells shown in e. Four fields were counted per condition and each condition was run in triplicate. The experiment was repeated twice and data from a representative experiment are shown. * *p* < 0.02 (*t*-test) comparing erlotinib+MLN0128 to vehicle.

To that end, we used IHC to measure the levels of POSTN in GBM tissues from treated mice. Figure 5a (upper panels) shows that POSTN expression was inhibited by the combination of erlotinib and MLN0128. In adjacent tissue sections, the number of Iba1-positive tumor-associated macrophages was also reduced by erlotinib+MLN0128 treatment (Figure 5a, lower panels). Iba1-positive cells respond to POSTN and CCL2 with increased migratory and pro-inflammatory phenotypes in vivo. Quantification of Iba1 positive cells in samples from mice treated with erlotinib, MLN0128, or the combination of the two drugs, shows that all three treatments inhibited the number of Iba1 positive cells infiltrating intracranial GBMs, with the combination being most effective (Figure 5b).

Since POSTN is a secreted cytokine, quantification of its IHC-detected levels is challenging. Therefore, we elected to validate the POSTN and CCL2 level changes, following treatment with erlotinib+MLN0128, in culture. We used GBM192 and GBM218, both of which harbor EGFR point mutations, amplification, and the presence of EGFRvIII (Table S1). Using commercially available ELISA assays, we measured the amount of POSTN and CCL2 secreted by the GBM PDXCs in the absence or presence of drug treatment over 48 h. As shown in Figure 5c, POSTN levels were inhibited by 50% in GBM192 and 40% in GBM218 following treatment with the combination of 20 µM erlotinib and 1 µM MLN0128 over 48 h. Similarly, CCL2 levels were significantly inhibited between 51% in GBM192 and 42% in GBM218 following treatment with erlotinib+MLN0128 (Figure 5d).

Cross-signaling between EGFR and EGFRvIII has been documented to contribute to GBM growth in vivo via increased CCL2 levels and activation of STAT3, a transcription factor enriched in the most aggressive mesenchymal subtype of GBMs [7,37,40]. Figure S4 shows that erlotinib+MLN0128 inhibited p-STAT3 in both GBM192 and GBM218, suggesting an additional potential anti-tumor mechanism underlying the effects of the dual therapy.

We next interrogated the functional significance of the regulation of POSTN levels by erlotinib and MLN0128. We used a “surrogate” human macrophage cell line (U937; [41]), primed toward an M2 phenotype, and performed transwell migration assays using chemo-attractants supernatants from GBM192 and GBM218 treated with erlotinib, MLN0128, or the combination of the two drugs. Representative photomicrographs of migrated U937 cells are shown in Figure 5e.

Quantitative analyses of migrated U937 cells shown in Figure 5f demonstrate that supernatant collected from GBM cultures treated with erlotinib+MLN0128 significantly inhibited migration of U937 cells by 62% (in the case of GBM192) and 55% (in the case of GBM218) compared to the vehicle. Supernatant collected from MLN0128-treated GBM cultures also inhibited U937 migration, albeit to a lesser extent—15% reduction in the case of GBM192 and 22% in the case of GBM218. These results suggest that the erlotinib+MLN0128 combination can effectively inhibit the infiltration of tumor-associated macrophages/microglia and thus exert additional anti-tumor activity in GBM.

## 4. Discussion

Precision medicine-guided individualized treatment of cancer has been implemented to date via clinical trials such as TAPUR or NCI-MATCH (NCT02693535; NCT02465060) targeting various solid tumors, however, these trials are not available for brain tumor patients. Thus, identifying effective therapeutic combinations for GBM patients remains an unmet and urgent clinical need. In this study, we set out to identify effective, individualized anti-tumor therapies for glioblastoma, using patient-derived GBM samples. We focused on the IDH1 wild-type, EGFR-altered GBMs because the patient outcome in this molecular subset of GBMs is significantly worse compared to GBMs with wild-type EGFR [7,30,34].

For drug screens, we selected agents for which evidence of blood–brain barrier penetrance exists either from clinical trials or from preclinical studies [42]. Our HTDS assays identified several combinations of EGFR+MTOR/AKT pathway inhibitors as effective at inhibiting the growth of EGFR-driven GBM samples, with erlotinib+MLN0128 ranking first (Figure 1e). Subsequent analyses uncovered tumor-cell-intrinsic mechanisms such as induction of apoptosis and downregulation of p-EGFR, p-AKT, p-ERK, and p-RAS40 levels, which underlie the synergistic anti-tumor activity of erlotinib+MLN0128. Importantly, the combination of erlotinib+MLN0128 showed significant anti-tumor activity in vivo, with prolongation of survival in tumor-bearing mice.

Using transcriptomic and proteomic approaches, we interrogated changes induced by each of the drugs alone and the combination thereof in tumor-driving signaling pathways. We document pharmacological inhibition of pro-inflammatory cytokines POSTN and CCL2 in the TME following combinatorial treatment of erlotinib+MLN0128 and propose that the effectiveness of the dual therapy is a result of both direct anti-tumor activity and modulation of the TME [5,9,16,43]. Of note, our in vivo studies were performed using Nu/Nu mice. Even though nude mice exhibit a limited immune cell repertoire, several studies have used this orthotopic glioma model to interrogate the GBM microenvironment in situ [41]. The TME has long been characterized as a critical regulator of tumor progression in GBM. Cellular composition, the origin of the TAMs, and the presence of immunosuppressive cytokines, together have been shown to contribute to therapy resistance, and glioma recurrence [35]. In addition, GBMs are characterized as “cold” tumors and so far immunotherapy using checkpoint inhibitors has not been effective [44].

CCL2 levels are anti-correlated with the survival of GBM patients, which is in part supported by the CCL2-induced M2 repolarization of macrophages in the TME [45]. While both POSTN and CCL2 have been previously implicated in promoting GBM aggressiveness [35,37,38], this is the first study that demonstrates effective pharmacological inhibition of these cytokines in vivo, using a combination of EGFR and MTOR-targeted drugs. Further, our results show that using rational combinations of clinically available drugs, we were able to effectively reduce the inflammatory GBM phenotype by decreasing the number of tumor-associated Iba1-positive macrophages/microglia.

The immune-modulatory effects of the dual therapy can be most directly attributed to the downregulation of pro-tumorigenic cytokines CCL2, CXCR4, and POSTN [46]. Inhibition of tumor-derived pro-inflammatory chemokines results in fewer immunosuppressive M2 macrophages, thus contributing to the anti-tumor effect noted in vivo. Our RNA profiling of the treated GBM tissues also identified the downregulation of CH3L1—specifically induced by the combination of erlotinib and MLN0128 in vivo (Figure 4a). In addition to its well-documented role in driving tumor angiogenesis [47], a recent study documented the role of CH3L1 in driving macrophage-mediated immunosuppression in GBM [48]. Thus, one additional potential mechanism of action underlying the immune-modulatory effects of the dual therapy may be due to the inhibition of CH3L1.

Our findings are both novel and translationally impactful, demonstrating that the combination of erlotinib and MLN0128 jointly inhibits tumor growth by targeting intrinsic tumor cell growth and cytokine secretion which in turn impacts the TME in vivo.

More broadly, our work demonstrates the feasibility of using the described translational oncology pipeline consisting of pharmacogenomic, individualized testing of clinical samples in conjunction with in vivo efficacy studies, to identify novel combinatorial therapies for GBM.

## 5. Future Remarks

Precision oncology approaches for GBM are yet to be systematically implemented in the clinic. The data we present herein support the concept of using personalized treatments for brain tumor patients, and specifically doing so based on individualized pharmaco-genomic testing in culture and in vivo, using patient-derived models of disease. Similar to the dual therapy of erlotinib and MLN0128 identified in this study, additional combinations of existing therapies can be explored and functionally tested, in an effort to offer more effective treatments for patients with GBM. Clinical trials testing such strategies would be the most obvious way to bring such findings to GBM patients [49].

## Figures and Tables

**Table 1:** Antibodies used for Western blot and immunohistochemical analyses.

Primary Antibody	Clone and Isotype	Dilution	Catalog Number	Supplier
Phospho-p44/42 MAPK (Erk1/2) (Thr202/Tyr204)	D13.14.4E; IgG	1:1000	4370S	Cell Signaling Technology
p44/42 MAPK (Erk1/2)	137F5; IgG	1:1000	4695S	Cell Signaling Technology
Phospho-Stat3 (Tyr705)	D3A7; IgG	1:500	9145S	Cell Signaling Technology
STAT3	polyclonal	1:1000	9132S	Cell Signaling Technology
Phospho-EGFR (Tyr1068)	polyclonal	1:500	44-788G	Invitrogen, Waltham, MA, USA
EGF Receptor (T43)	polyclonal	1:500	2963S	Cell Signaling Technology
Phospho-Akt (Ser473)	polyclonal	1:500	9271S	Cell Signaling Technology
Akt	polyclonal	1:1000	9272S	Cell Signaling Technology
EGFRvIII	DH8.3	1:500	NBP2-50599	Novus Biologicals, Centennial, CO, USA
GAPDH	6C5, IgG1	1:5000	MAB374	Millipore-Sigma, Burlington, MA, USA

## Data Availability

The microarray data associated with this study are freely available on the NCBI/GEO site, under accession number GSE178147.

## Supplementary Materials

The following are available online at https://www.mdpi.com/article/10.3390/cells12040547/s1, Figure S1: Quantification of Western blot analyses shown in Figure 2, Figure S2: Effects of erlotinib and MLN018 in recurrent GBM212, Figure S3: Apoptosis assays in GBM 192 and GBM218, Figure S4: p-STAT3 regulation in GBMs with EGFRvIII, Table S1: Clinical and genomic annotations for ten GBM samples included in this study, Table S2: Ranking of single drugs in GBM180. The table includes the list of individual drugs and their respective molecular targets, as well as the AUCs and ranking scores, following HTDS of GBM180, Table S3: Ranking of combination treatments in GBM180. The table includes each combination shown in the heatmap (Figure 1d) and the viability scores measured when drugs were used at 10% Cmax. Scores are shown from low to high (in descending order of anti-tumor efficacy), Table S4: Scores of drugs and combinations used for screening ten EGFR-driven GBM samples. The table shows the scores for each drug and combination shown in Figure 1e when used at 10% Cmax in 10 GBM samples displayed in the heatmap, Table S5: Differentially expressed genes in GBM192 treated in vivo with erlotinib, MLN0128, and the erlotinib+MLN0128. The table includes all DEG obtained after microarray analysis of RNA samples from treated mice. The analysis strategy is described in the methods section. Fold change (Log2) values and adjusted p values are shown for all three comparisons. TRE1 is erlotinib, TRE2 is MLN0128, TRE3 is the combination of the two drugs, and V is the vehicle. Genes are annotated showing ensemble gene, transcript, and protein ID. The ID column refers to the microarray probe used to detect each specific transcript.
