# Supplementary material for: Dual Targeting of EGFR and MTOR Pathways Inhibits Glioblastoma Growth by Modulating the Tumor Microenvironment
Source: Cells. 2023 Feb 8;12(4):547. doi: 10.3390/cells12040547 (PMC9954001; doi:10.3390/cells12040547)
Supplement: Supplementary file 1 [file cells-12-00547-s001.zip › cells-2174606 supplementary Figures.pdf]

# Supplementary Materials

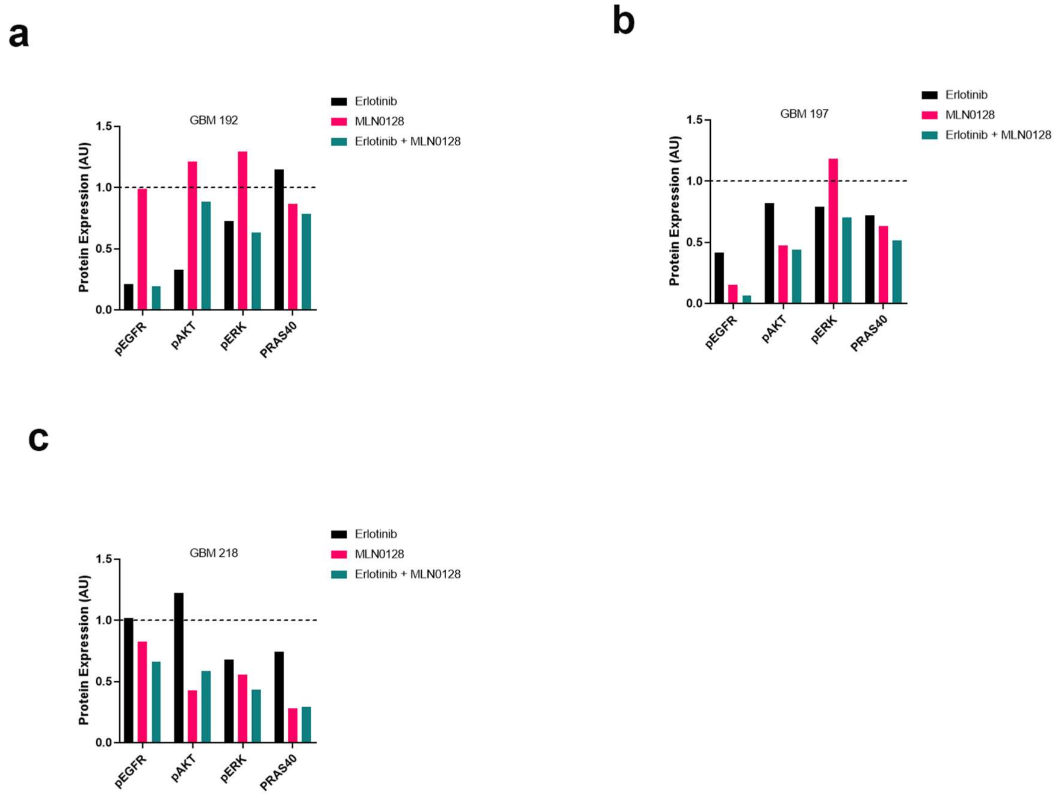


**Figure S1: Quantification of Western blot analyses shown in Figure 2 c-e.** Image J software was used to measure the pixel density of each band. Normalization was done to GAPDH levels and then to the levels of total protein for each phosphoprotein shown. Data is shown as fold change from the levels of the same phosphoprotein in the vehicle-treated sample (set at 1 and indicated by the dotted line). **a**. Quantification of indicated phosphoproteins following treatment with erlotinib, MLN0128 or the combination of both drugs in GBM192. **b**. Same quantification for GBM 197. **c**. Same quantification for GBM218.

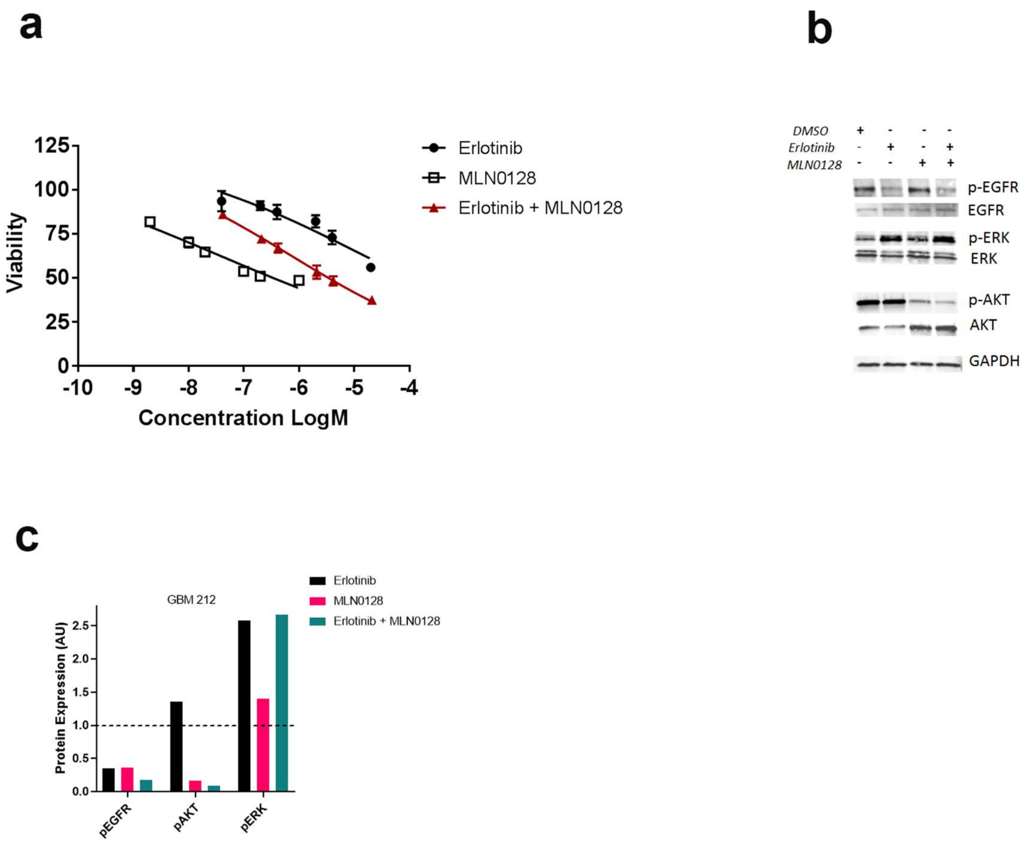


**Figure S2: Effects of erlotinib and MLN018 in recurrent GBM212. a.** Full dose response effects on cell viability in GBM212 treated with erlotinib, MLN0128 or the combination of the two drugs. **b**. Western blot analyses of p-EGFR, p-AKT and p-ERK in samples of GBM212 treated for 24 hours as indicated. Total EGFR, AKT and ERK are also shown. GAPDH was used as a loading control. **c**. Quantification of phosphoprotein levels from panel b was done as explained in Figure S1.

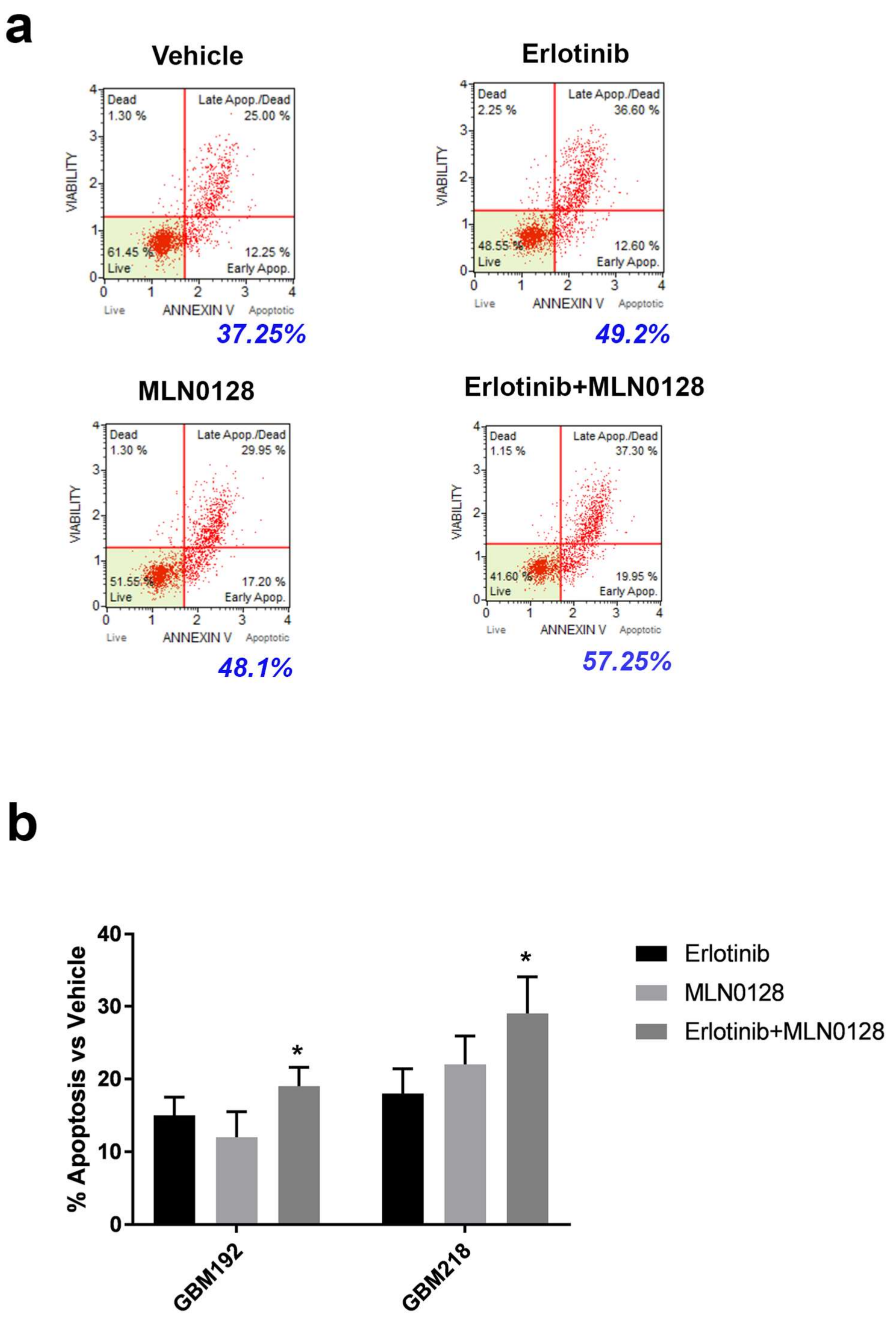


**Figure S3: Apoptosis assays in GBM 192 and GBM218. a.** Caspase 3/7 assays were run using GBM samples treated for 48h with erlotinib (20mM), MLN0128 (1mM) or the combination of the two drugs, as recommended by the manufacturer of the kit. Representative screenshots for each treatment condition are shown for GBM192. **b**. Quantification of apoptotic cells in GBM192 and GBM218. Each sample was run in triplicate and the experiment was repeated twice for each cell line. * $p<0.05$, t-test comparing the total number of apoptotic cells in the combination treatment vs vehicle.

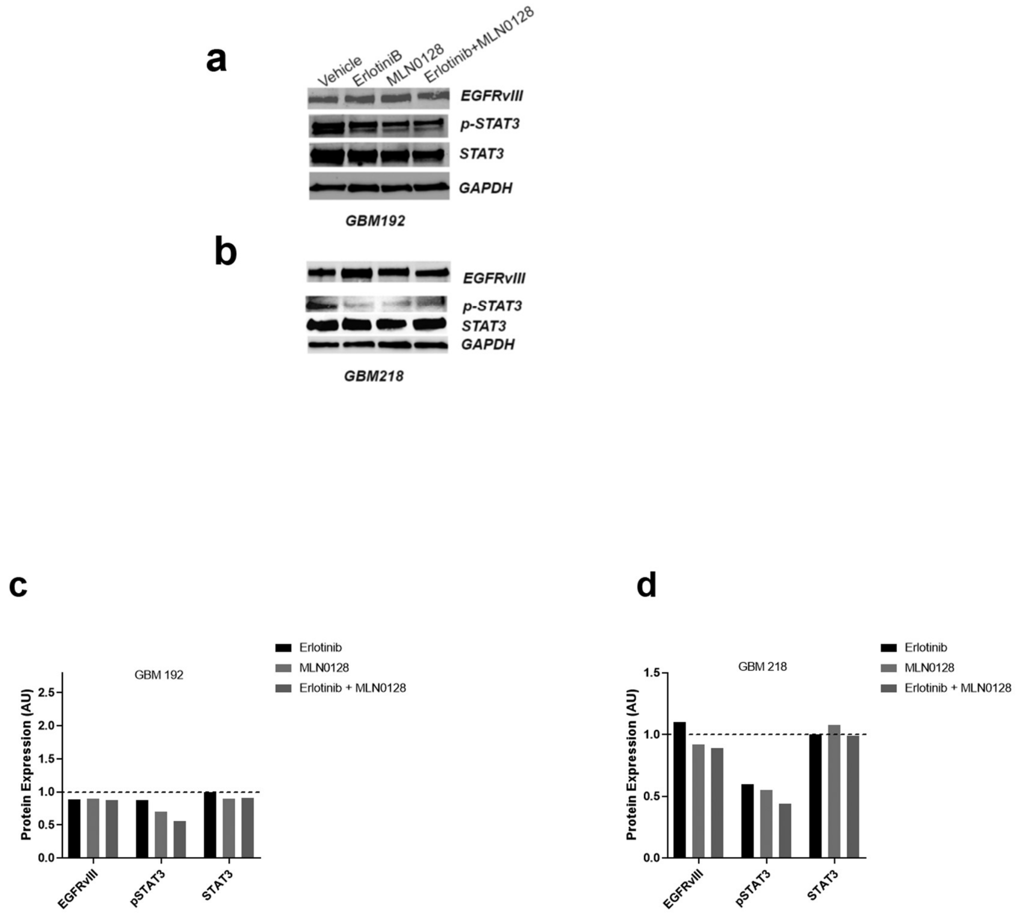


**Figure S4: p-STAT3 regulation in GBMs with EGFRvIII. a.** Western blot analyses of EGFRvIII, p-STAT3 and STAT3 in GBM192 following treatment with erlotinib (20mM), MLN0128(1mM), or the combination of the two drugs for 24h. GAPDH was used as a loading control. **b**. Western blot analyses of EGFRvIII, p-STAT3 and STAT3 in GBM218 following treatment with erlotinib (20mM), MLN0128(1mM), or the combination of the two drugs for 24h. GAPDH was used as a loading control. **c**-**d**. Quantification of protein levels shown in a-b. The quantification was performed as explained in Figure S1.
